# Photo-Charging of Li(Ni_0.65_Co_0.15_Mn_0.20_)O_2_ Lithium-Ion Battery Using Silicon Solar Cells

**DOI:** 10.3390/ma15082913

**Published:** 2022-04-15

**Authors:** Seungbum Heo, Baeksang Yoon, Hyunsoo Lim, Hyung-Kee Seo, Cheul-Ro Lee, Inseok Seo

**Affiliations:** 1 Department of Electrical Engineering, Hanbat National University, Daejeon 34158, Korea; heosb3@ex.co.kr; 2 School of Advanced Materials Engineering, Jeonbuk National University, Baekje-daero 567, Jeonju 54896, Korea; qortkd2134@jnbu.ac.kr (B.Y.); kady1004@naver.com (H.L.); crlee7@jbnu.ac.kr (C.-R.L.); 3 Future Energy Convergence Core Center, School of Chemical Engineering, Jeonbuk National University, Baekje-daero 567, Jeonju 54896, Korea; hkseo@jbnu.ac.kr

**Keywords:** Li(Ni_0.65_Co_0.15_Mn_0.20_)O_2_, Li_4_Ti_5_O_12_, lithium-ion battery, silicon solar cell, integrated device

## Abstract

This study reports an integrated device in which a lithium-ion battery (LIB) and Si solar cells are interconnected. The LIB is fabricated using the Li(Ni_0.65_Co_0.15_Mn_0.20_)O_2_ (NCM622) cathode and the Li_4_Ti_5_O_12_ (LTO) anode. The surface and shape morphologies of the NCM and LTO powders were investigated by field emission scanning electron microscopy (FE-SEM). In addition, the structural properties were thoroughly examined by X-ray diffraction (XRD). Further, their electrochemical characterization was carried out on a potentiostat. The specific discharge capacity of the NCM cathode (half-cell) was 188.09 mAh/g at 0.1 C current density. In further experiments, the NCM-LTO full-cell has also shown an excellent specific capacity of 160 mAh/g at a high current density of 1 C. Additionally, the capacity retention was outstanding, with 99.63% at 1 C after 50 cycles. Moreover, to meet the charging voltage requirements of the NCM-LTO full-cell, six Si solar cells were connected in series. The open-circuit voltage (V_OC_) and the short-circuit photocurrent density (J_SC_) for the Si solar cells were 3.37 V and 5.42 mA/cm^2^. The calculated fill factor (FF) and efficiency for the Si solar cells were 0.796 and 14.54%, respectively. Lastly, the integrated device has delivered a very high-power conversion-storage efficiency of 7.95%.

## 1. Introduction

Environmental problems caused by fossil fuels, such as greenhouse gas (GHG) emissions, drought, and rising sea levels, have recently become major global issues [1,2]. Therefore, many studies have investigated eco-friendly renewable energy sources (solar, wind, geothermal, etc.) that generate electricity with a minimal carbon footprint [3]. Solar energy systems have attracted the most attention among these renewable energy sources as the sun is a popular infinite free energy source. Therefore, photovoltaics (PV) have the potential to meet the global energy demand [4]. However, the intermittent availability of sunlight is a significant drawback that hinders its various applications. In the present study, the idea is to connect the solar cell to LIB directly without any converter. The integration of energy generation and energy storage systems has been presented as one of the better solutions to address the intermittent nature of sunlight [5]. Liao et al. reported the need for a bidirectional DC/DC converter to control a solar power system [6]. The bidirectional DC/DC converter system was more complex and expensive than the direct connection of the solar cell and the energy storage device. Therefore, a direct DC storage system is currently in the spotlight by using the integrated device as a promising technology. Many studies have used capacitors and lithium-ion batteries (LIBs) as storage for charging by PVs [7,8,9,10,11].

LIB is one of the potential energy storage devices used in a wide range of applications, from miniaturized devices to high-power-driven electric vehicles. They are considered suitable energy storage devices that can be charged by solar-powered PVs. It is known that the electrochemical performance of LIB is mainly dependent on the active cathode and anode electrode materials. Among the LIB cathode materials, the LiFePO_4_ (LFP) cathode material has been widely used in energy storage systems (ESS) because of its low price and high safety. However, LFP has an inferior specific capacity compared to the Li(Ni_x_Co_y_Mn_z_)O_2_ (NCM) cathode material [12,13,14]. This study proposes to use the NCM622 cathode material. This cathode material is richer in nickel content than NCM333, commonly used in the past, which results in high discharge capacity and energy density.

Moreover, the lower cobalt (Co) content reduces the overall cost of the batteries [15]. On the other hand, Li_4_Ti_5_O_12_ (LTO) is preferred among the anode materials due to its high-capacity retention rate with a reasonable specific capacity [16]. In addition, LTO also has a stable voltage plateau at 1.5 V vs. Li/Li^+^. This high-voltage plateau of LTO can prevent the reduction of electrolytes and can do better than the Li metal anode. Therefore, NCM622 and LTO are chosen to increase the specific capacity and achieve high cycle retention. In this study, as discussed earlier, an integrated device is designed by combining LIB and Si solar cells without any converter. The morphological and structural characterization of the NCM622 cathode powder and the LTO anode powder were evaluated by FE-SEM and XRD, respectively. The electrochemical properties were obtained from the NCM (NCM vs. Li/Li^+^) half-cell, LTO (LTO vs. Li/Li^+^) half-cell, and NCM-LTO full-cells. Later, six single Si solar cells were connected in series to obtain sufficient voltage to charge the battery. The electrical properties such as open-circuit voltage, short-circuit photocurrent density, fill factor, and efficiency of Si solar cells were analyzed with a solar simulator. Finally, an integrated device was fabricated by directly combining Si solar cells and an NCM-LTO full-cell. Furthermore, the photovoltaic charging of the NCM-LTO full-cell was successfully demonstrated.

## 2. Materials and Methods

### 2.1. Preparation of LIB and Solar Cell

The cathode slurry was prepared by dispersing NCM powder (active material), carbon black (super P), and polyvinylidene difluoride (PVDF) in N-Methyl-2-pyrrolidone (NMP) solvent at a respective weight ratio of 8:1:1. Similarly, the anode slurry was prepared using LTO powder as the active material. The mass loading of cathode and anode material was 7.59 and 8.78 mg/cm^2^, respectively. Additionally, the densities of the cathode and anode were 3.05 and 2.61 g/cm^3^, respectively. The cathode and anode slurries were coated on the aluminum foil and the copper foil, respectively. The coated slurries were then dried and pressed to make sheet electrodes. The half-cells and full-cells were assembled in a glove box filled with argon gas (H_2_O < 0.01 ppm, O_2_ < 0.01 ppm), and 1.2 M LiPF_6_ in a 1:1 (*v*/*v*) solution of ethylene carbonate and dimethyl carbonate was used as an electrolyte. Then, 150 μL of electrolyte was added for coin cells. Li metal foil with a thickness of 0.45 mm was used for coin cell fabrication (MTI Korea). The half-cell was fabricated in the form of the coin cell (CR 2032), while the full-cell was assembled in the form of a pouch (4.5 × 4.5 cm). For the pouch cell, nickel and aluminum were used as the cathode and anode lead tabs, and 400 μL of electrolyte was added. The loading and density of electrodes had the same value as the coin cell fabrication. Polyethylene separator was used for every cell fabrication. The electrochemical performance data of half-cells and full-cells corresponds to the coin cell and pouch cell configuration, respectively. A polycrystalline Si solar cell with six single Si solar cells (SAVE SOLAR Co. Ltd. Washington DC, USA) connected in series was integrated with LIB. Therefore, it provides the required voltage for LIB charging.

### 2.2. Characterizations

The NCM622 precursor was prepared by the co-precipitation method. To improve the packing density of the cathode electrode, the NCM powder was a mixture of large and small particles in a 7:3 ratio. Commercial LTO powder was purchased from Sigma Aldrich Co. Ltd St. Louis, MO, USA. The surface and shape morphologies of the NCM and LTO powders were characterized by field emission scanning electron microscopy (FE-SEM) (SU-70 Hitachi High-Tech Corporation, Fukuoka, Japan). The acceleration voltage for the FE-SEM measurement was 10 kV. The crystal structures were analyzed using X-ray diffraction (XRD) (MAX-2500, RIGAKU, Tokyo, Japan) with Cu-kα radiation over a 2θ range of 10–80°, with a step size of 0.05° and a scan rate of 2.0°/min. The electrochemical properties of the NCM and LTO half-cells and NCM-LTO full-cell were evaluated using a battery cycler (WBCS3000S, WONATECH Co. Ltd., Seoul, Korea). Galvanostatic charge–discharge tests of the NCM-LTO full-cell were conducted in the 1.6–3.0 V potential window. The NCM-LTO full-cell was tested for rate capability at different current densities. Further, cyclic stability studies were performed on the NCM-LTO full-cell (pouch cell configuration) for 50 cycles at 1 C. The open-circuit voltage (V_OC_), short-circuit photocurrent density (J_SC_), fill factor (FF), and efficiency were measured by irradiating Si solar cells with 100 mW/cm^2^ light with a solar simulator (PEC-L01, Peccell Technologies, Yokohama, Japan). All tests were performed at room temperature.

### 2.3. Integrated Si Solar Cells–LIB Device

The integrated device was fabricated by directly connecting the Si solar cells and LIB. The LIB was charged by irradiating light to Si solar cells using a solar simulator (PEC-L01, Peccell Technologies, Yokohama, Japan). Before testing, the light intensity was calibrated to 100 mW/cm^2^. The Si solar cells were photo-charged using a solar simulator then discharged with a battery cycler.

## 3. Results and Discussion

**Figure 1:**
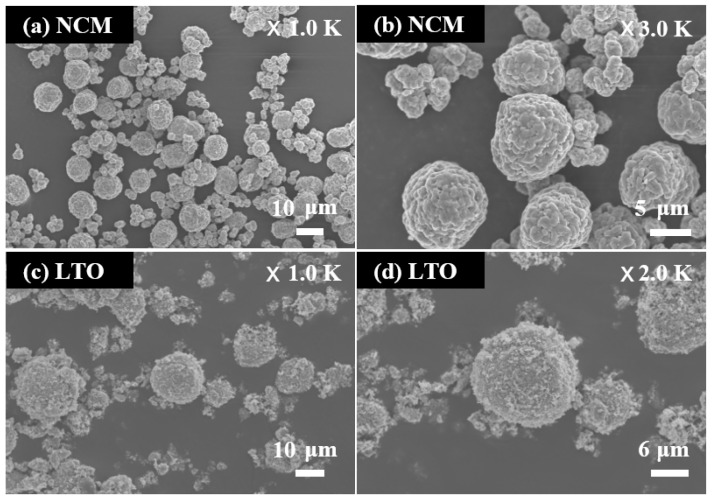
FE-SEM images of (**a**,**b**) NCM powder and (**c**,**d**) LTO powder.

Figure 1 displays the FE-SEM images of the NCM and LTO powders. The particle size and shape of the NCM powder are shown in Figure 1a,b. Figure 1a shows that NCM powder was composed of large and small particles. The NCM powder consists of large and small particles with respective sizes of about 10 and 3 μm; additionally, the large and small particles were mixed in an optimized 7:3 weight ratio, respectively. The optimization was performed with an objective to improve the high-energy density [17]. Figure 1b shows the agglomerated particles of NCM powder. Submicron particles were aggregated to form large and small particles. Figure 1c,d illustrate the particle morphology of LTO powder. Figure 1c shows that the LTO powder contains porous spherical particles with a diameter of about 20 μm. The spherical shape improves the high rate performance of the LTO by minimizing the Li-ion diffusion path lengths [18]. Figure 1d shows a detailed view of the porous spherical particles of LTO powder. The porous spherical particles are made from aggregated particles with a size of less than about 0.5 μm.

**Figure 2:**
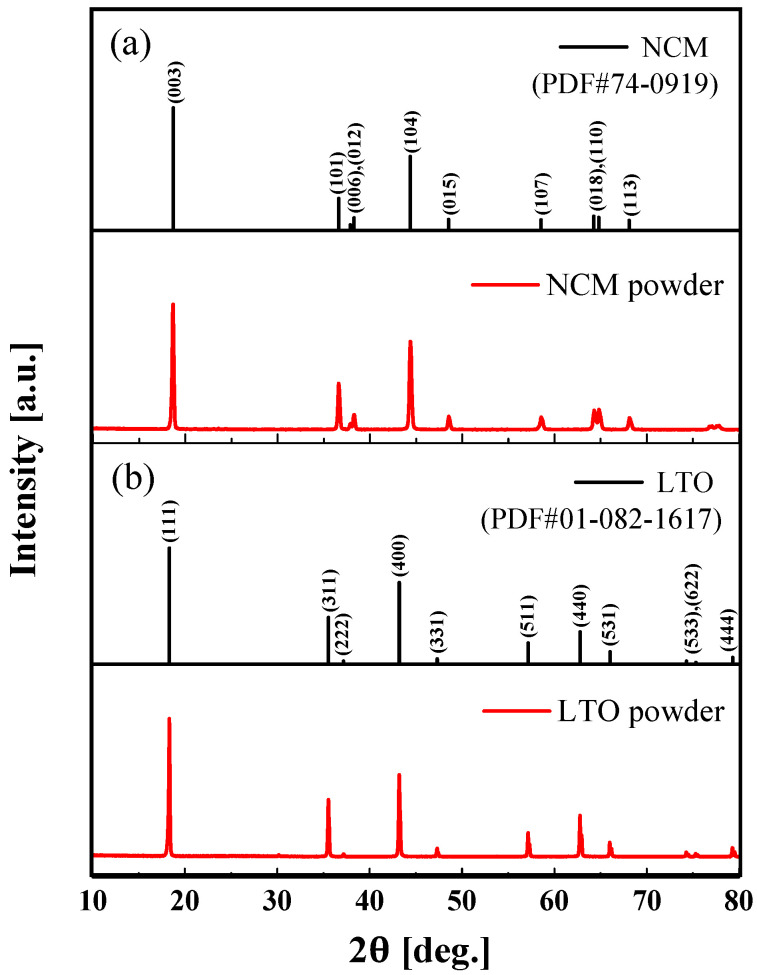
XRD patterns of (**a**) NCM (PDF#74-0919) powder and (**b**) LTO (PDF#01-082-1617) powder.

Figure 2a,b, respectively, depict the XRD patterns of the NCM and LTO powders. As shown in Figure 2, the XRD patterns of NCM and LTO powder were identified and indexed with the powder diffraction database (PDF). The XRD peaks of NCM powders around 18.7°, 36.6°, 37.9°, 38.7°, 44.4°, 48.5°, 58.5°, 64.3°, 64.8°, and 68.1° are assigned to the (0 0 3), (1 0 1), (0 0 6), (0 1 2), (1 0 4), (0 1 5), (1 0 7), (0 1 8), (1 1 0), and (1 1 3) planes, respectively. The diffraction peaks of LTO powders around 18.3°, 35.5°, 37.1°, 43.2°, 47.3°, 57.1°, 62.7°, 66.0°, 74.2°, 75.2°, and 79.2° are assigned to the (1 1 1), (3 3 1), (2 2 2), (4 0 0), (3 3 1), (3 3 3), (4 4 0), (5 3 1), (5 3 3), (6 2 2), and (4 4 4) planes, respectively. The XRD measurement results showed that the NCM and LTO powders were successfully synthesized.

**Figure 3:**
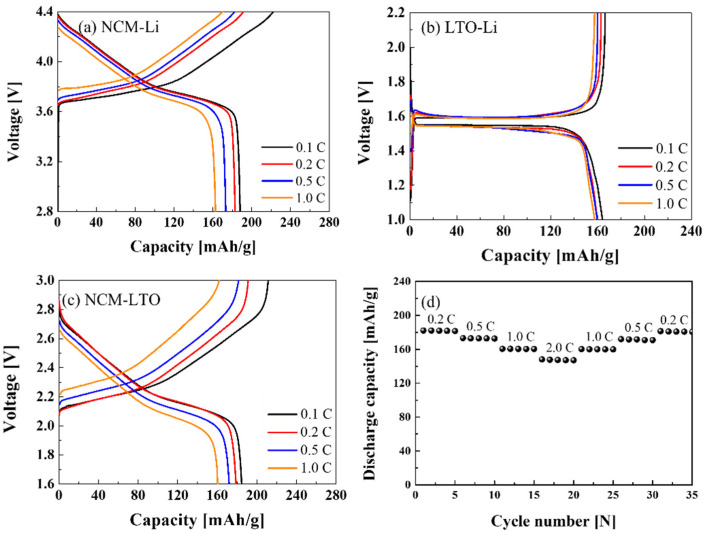
Electrochemical properties of (**a**) NCM-Li, (**b**) LTO-Li, and (**c**) NCM-LTO full-cells, and (**d**) rate capability of the NCM-LTO full-cell at various C-rates.

Figure 3a–c show the electrochemical properties at 0.1, 0.2, 0.5, and 1 C of the NCM-Li, LTO-Li half-cells, and the NCM-LTO full-cell, respectively. Figure 3a reveals the discharge capacities of 188.09, 182.92, 173.27, and 162.81 mAhg^−1^ at 0.1, 0.2, 0.5, and 1 C, respectively. The discharge capacities of the NCM-Li half-cell were similar to those of reported values in the previous study [19]. Figure 3b shows that the discharge capacities of LTO-Li half-cells were 161.3, 159.76, 159.7, and 157.57 mAhg^−1^ at 0.1, 0.2, 0.5, and 1 C, respectively. Further, the NCM-LTO full-cell has delivered a stable specific discharge capacity of 185.79 mAhg^−1^ at 0.1 C, 182.02 mAhg^−1^ at 0.2 C, 173.19 mAhg^−1^ at 0.5 C, and 160.48 mAhg^−1^ at 1 C. The discharge capacity of the NCM-LTO full-cell was very similar to that of the NCM-Li half-cell. Additionally, the operating voltage of the full-cell was near about 2.3 V. This operating voltage is reasonable for integrating solar cells with the battery. Figure 3d shows the discharge capacities of the NCM-LTO full-cell at various C-rates. The NCM-LTO full-cell showed the same discharge capacities during five cycles at the same C-rate, which supports the stable performance of the NCM-LTO full-cell.

**Figure 4:**
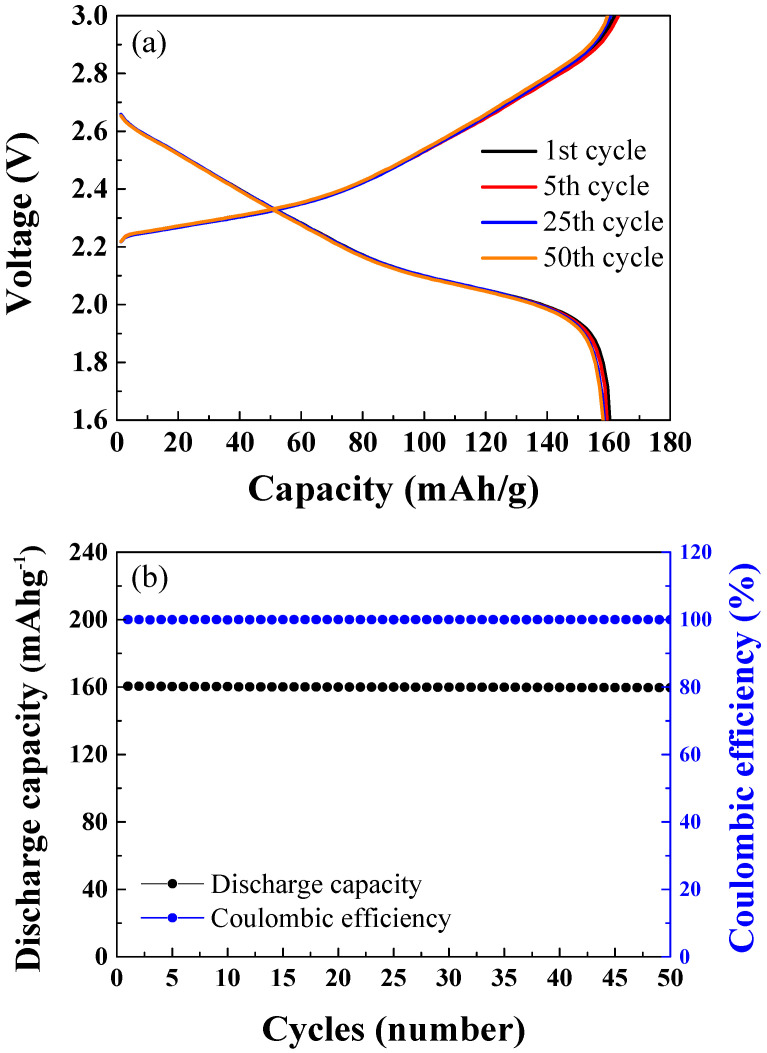
(**a**) Cycle performance of the NCM-LTO full-cell over 50 cycles. (**b**) Discharge capacity and coulombic efficiency of the NCM-LTO full-cell over 50 cycles.

Figure 4a,b show the results of cycle tests for 50 cycles of the NCM-LTO full-cell at 1 C-rate. The charge–discharge was carried out in the 1.6–3.0 V range. The discharge capacities were 160.48 mAhg^−1^ at the 1st cycle, 160.32 mAhg^−1^ at the 5th cycle, 159.97 mAhg^−1^ at the 25th cycle, and 159.89 mAhg^−1^ at the 50th cycle. The NCM-LTO full-cell exhibited excellent cycle retention of 99.63% even after 50 cycles. Figure 4b shows the discharge capacities and coulombic efficiency with respect to the cycle number for 50 cycles. In cycles 1, 5, 25, and 50, the coulombic efficiencies were 99.99%, 99.98%, 99.97%, and 99.95%, respectively. This shows the exceptional stability of the electrode materials.

**Figure 5:**
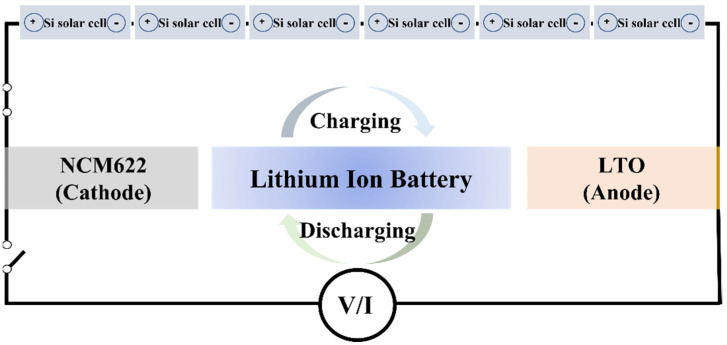
Schematic diagram of the NCM-LTO integrated device with Si solar cells.

Figure 5 shows a schematic diagram of the integrated device using the Si solar cell and the NCM-LTO full-cell. The voltage range of the NCM-LTO full-cell was 1.6–3.0 V. The necessary charging voltage was obtained by connecting six polycrystalline single Si solar cells of 0.57 V each in series. The integrated device was fabricated by connecting directly without a converter. Using this system, the integrated device can charge the battery through photo-charging.

**Figure 6:**
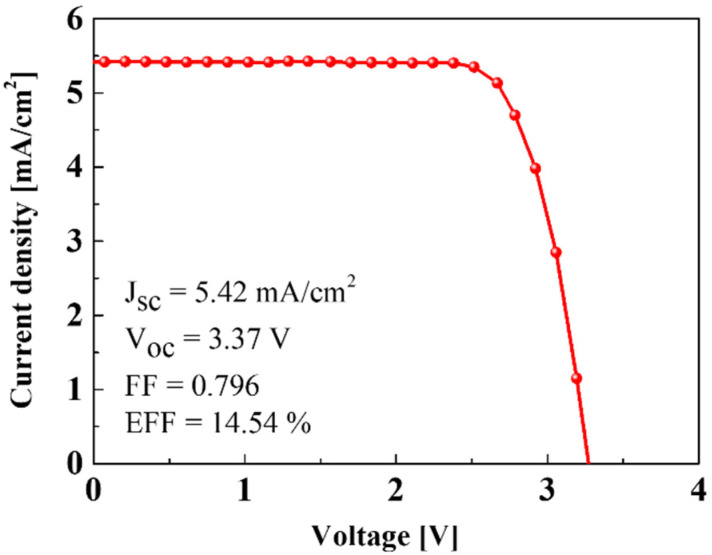
J-V curve of the Si solar cells.

Figure 6 depicts the current density-voltage (J-V) characteristics of the interconnected Si solar cells. The short-circuit photocurrent density (J_SC_) and open-circuit voltage (V_OC_) of a Si solar cell were 5.42 mA/cm^2^ and 3.37 V. The interconnected Si solar cells can provide the required voltage to charge the NCM-LTO full-cell. The fill factor (FF) and efficiency of the Si solar cells were calculated to be 0.796 and 14.54%, respectively. The Si solar cells used in this study have displayed a slightly higher efficiency of 14.54%, compared to commercially available Si solar cells, which are typically about 12–15% [20].

**Figure 7:**
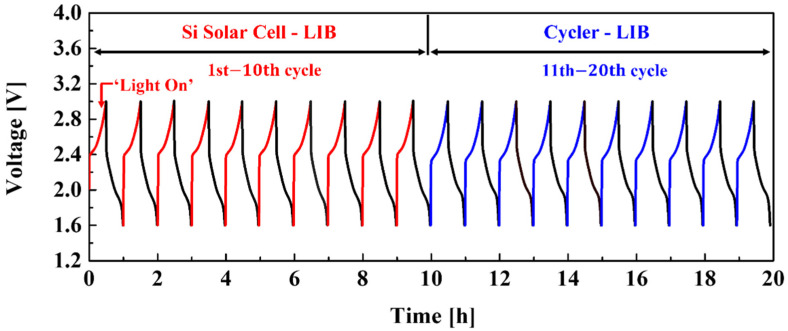
Photo-charging and discharging diagram of the NCM-LTO full-cell using the Si solar cell.

Figure 7 shows the electrochemical results of the integrated device. The charge–discharge tests were carried out for 20 cycles in the 1.6–3.0 V range. The integrated device was charged by the Si solar cells using a solar simulator and discharged by a battery cycler to the 10th cycle. After that, the NCM-LTO full-cell was charged and discharged for 11 to 20 cycles using a battery cycler. The current density of the integrated device was 40 mA. The photo-charging and galvanostatic discharging profiles from 1 to 10 cycles were similar to those of galvanostatic charge and discharge (using battery cycler) from 11 to 20 cycles. The power conversion/storage efficiency of the integrated device was 7.95%. Moreover, energy storage efficiency from the Si solar cells to the NCM-LTO full-cell was 54.36%. Low-power conversion efficiency has represented an essential obstacle in researching integrated systems wherein Si solar cells and batteries are connected. It is expected that the integrated device fabricated in this study can figure out the issues of low-power conversion efficiency [10].

**Figure 8:**
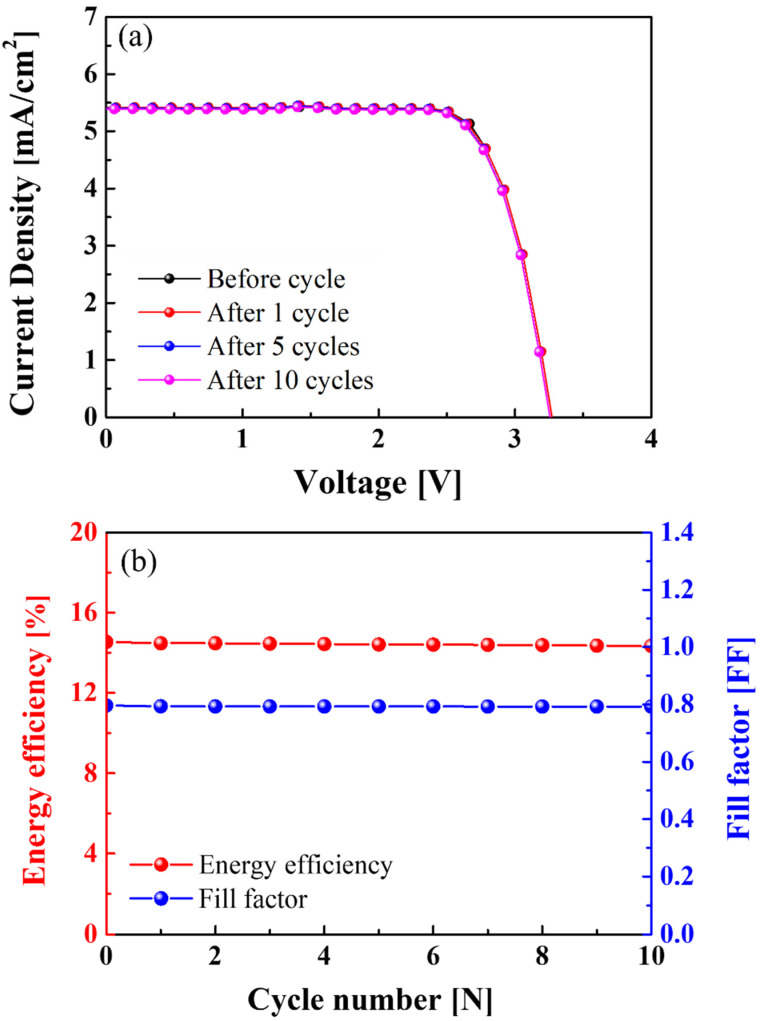
(**a**) J-V curve and (**b**) energy efficiency and fill factor (FF) of Si solar cells connected to the NCM-LTO full-cell before and after 10 cycles.

Figure 8a shows the J-V characteristics of the Si solar cells connected to the NCM-LTO full-cell before cycle testing and after 1st, 5th, and 10th cycle tests. The open-circuit voltage (V_OC_) was 3.37 V before the cycle test, and 3.37, 3.36, and 3.35 V during the 1st, 5th, and 10th cycles, respectively. The short-circuit photocurrent density (J_SC_) has remained constant with increasing cycle numbers. The J_SC_ was 5.42 mA/cm^2^ before the cycle test, and 5.42, 5.41, and 5.4 mA/cm^2^, during the 1st, 5th, and 10th cycles, respectively. Figure 8b shows the energy efficiency and fill factor (FF) of the Si solar cells connected to the NCM-LTO full-cell before and after various cycle tests. Before cycle testing, the fill factor (FF) was 0.796. Then, the FF was 0.793, 0.793, and 0.792 at the 1st, 5th, and 10th cycles, respectively. These results show that the FFs were almost unchanged during the test. The efficiency was 14.54% before the cycle test, and after the 1st, 5th, and 10th cycle tests, it slightly reduced to 14.48%, 14.42%, and 14.35%, respectively. The energy conversion efficiency of the Si solar cells connected to the NCM-LTO full-cell was 98.69% before and after 10 cycles. Therefore, the Si solar cells were suitable as a device for supplying power to the high-performance NCM-LTO full-cell and can pave the way to futuristic energy-saving technologies.

## 4. Conclusions

The NCM-LTO full-cell was fabricated using NCM and LTO as the cathode and the anode, respectively. The as-fabricated full-cell has delivered a high discharge capacity and stable performance, with 99.63% cycle retention after 50 cycles. This high performance is mainly due to the synergy of the optimized NCM622 cathode and LTO anode materials compared to conventional LFP-graphite batteries. Besides, the NCM-LTO full-cell is directly interconnected with six Si solar cells without any convertor. The integrated device was photo-charged by the Si solar cell and galvanostatic discharged by a cycler. The power conversion efficiency of the NCM-LTO full-cell and Si solar cells was 7.95%, while the energy conversion efficiency reached 54.36%. Therefore, the present integrated system using the NCM-LTO full-cell could be a promising technology for the next-generation self-charging devices.

## Data Availability

The data presented in this study are available on request from the corresponding author. The data are not publicly available as it is part of the ongoing project.
